# Gripping performance in the stick insect *Sungaya inexpectata* in dependence on the pretarsal architecture

**DOI:** 10.1007/s00359-022-01570-1

**Published:** 2022-09-24

**Authors:** Julian Winand, Stanislav N. Gorb, Thies H. Büscher

**Affiliations:** Department of Functional Morphology and Biomechanics, Zoological Institute of the University of Kiel, Am Botanischen Garten 1–9, 24118 Kiel, Germany

**Keywords:** Phasmatodea, Locomotion, Attachment pad, Claws, Arolium, Holding forces

## Abstract

Insect attachment devices and capabilities have been subject to research efforts for decades, and even though during that time considerable progress has been made, numerous questions remain. Different types of attachment devices are known, alongside most of their working principles, however, some details have yet to be understood. For instance, it is not clear why insects for the most part developed pairs of claws, instead of either three or a single one. In this paper, we investigated the gripping forces generated by the stick insect *Sungaya inexpectata*, in dependence on the number of available claws. The gripping force experiments were carried out on multiple, standardized substrates of known roughness, and conducted in directions both perpendicular and parallel to the substrate. This was repeated two times: first with a single claw being amputated from each of the animals’ legs, then with both claws removed, prior to the measurement. The adhesive pads (arolia) and frictional pads (euplantulae) remained intact. It was discovered that the removal of claws had a detractive effect on the gripping forces in both directions, and on all substrates. Notably, this also included the control of smooth surfaces on which the claws were unable to find any asperities to grip on. The results show that there is a direct connection between the adhesive performance of the distal adhesive pad (arolium) and the presence of intact claws. These observations show collective effects between different attachment devices that work in concert during locomotion, and grant insight into why most insects possess two claws.

**Supplementary Information:**

The online version contains supplementary material available at 10.1007/s00359-022-01570-1.

## Introduction

Legged locomotion of insects has attracted significant interest from researchers over the decades. Functional morphology of their extremities and associated structures (Cruse 1976; Gorb 1996, 2001, 2005; Federle et al. 2001), as well as their locomotion control mechanisms (Koditschek et al. 2004; Ijspeert 2008) are well studied and also contributed to bionics engineering (biomimetics) and modern robotics, in particular soft robotics (Kim et al. 2013; Li et al. 2016; Shintake et al. 2018). Examples include a biomimetic robot based on both insect leg configuration (hexapodic) as well as their neuronal control mechanism (Bal 2021), hexapods based on soft robotic dielectric elastomer actuation (DEA) (Nguyen et al. 2018) and a robot with a beetle claw inspired locomotion system using open and closed claw states for smooth and slipless walking (Shima et al. 2020). Climbing robots such as one based on insect claws and tarsal spines (Liu et al. 2019) have also been developed. Claws are not the only pretarsal attachment devices of insects, but the most relevant for attachment to rough substrates (Song et al. 2016). Functionally, claws can be extensively modified to some specialized functions, especially, if the animal they are located on inhabits special habitats, such as in parasites which are adapted to their host surfaces (Büscher et al. 2022; Petersen et al. 2018). However, the majority of insects possesses pointed claws with one tip. Research on the claws ranges from experiments on the use and function of species of various taxa, like for example true bugs (Salerno et al. 2018), beetles (Bullock and Federle 2011; Voigt et al. 2019; Salerno et al. 2022), stick insects (Büscher et al. 2020), and flies (Salerno et al. 2020), including locomoting insects, and even their exuviae (Büsse et al. 2019). Furthermore there are investigations on the material properties of the claws (Li et al. 2022). In general, most of these studies show that the performance of the claw is mostly influenced by the roughness of the surface and that the interlocking requires larger surface asperities than the claw tip radius (Song et al. 2016). However, it has also been shown that this relationship between claw size and surface roughness is subject to the different size of the insects, or their claws respectively, both across species and within the same during growth (Pattrick et al. 2018).

Phasmatodea (stick and leaf insects) have been a model system for investigation of insect locomotion for several decades as well as a blueprint for robotic design. They are a widespread, herbivorous group of large terrestrial insects strongly adapted to foraging on plants (Bradler et al. 2014; Bradler and Buckley 2018). The plant structure and surfaces are extremely diverse and this has resulted in the evolution of highly adaptable attachment structures suited for different plant substrates (Büscher et al. 2018). Phasmids in particular possess claws used for mechanical interlocking with rough surface features, pretarsal arolia used to resist pull-off forces on smooth and micro-rough substrates, and tarsal euplantulae that generate friction and resist shear forces (Labonte and Federle 2013). The phasmatodean claws serve to grip on medium rough to very rough surfaces (Dai et al. 2002), where they mechanically interlock with asperities limited by their tip sharpness. They constitute the dominant contribution to attachment performance on surfaces with features sizes in the range of claw tip diameter to claw height. On either much smaller or much larger feature sizes of the substrate surface, their performance quickly diminishes (Song et al. 2016). Phasmatodean pretarsal adhesive pads (arolia) mainly interact with smooth surfaces, where they are able to form an intimate contact and by this enhance adhesion and friction (Büscher and Gorb 2021). They always generate a certain degree of attachment, but are mainly used on surfaces, where the claws do not find grip on, by this effectively extending the range of substrates for locomotion (Song et al. 2016). Phasmatodean euplantulae or tarsal pads (euplantulae) partially function in a similar way to the pretarsal adhesive pads. They can be hairy or smooth, segmented or otherwise structured, and can support different modes of locomotion and clinging. Euplantulae mainly act under compression and are used to counter shear forces and stabilize postures (Labonte et al. 2014, 2019; Federle and Labonte 2019; Büscher and Gorb 2019).

**Fig. 1:**
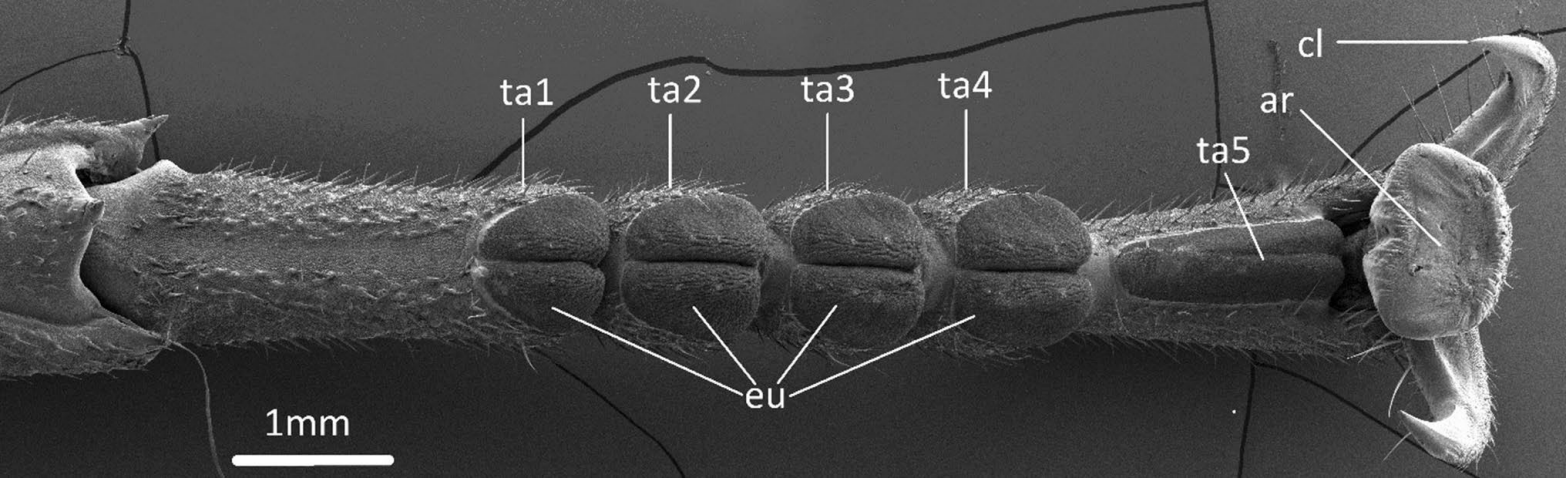
Representative phasmatodean tarsus from Sungaya inexpectata. cl, tarsal claw; ar, arolium; eu, euplantulae; ta1-5, tarsomeres 1–5

These different attachment devices (arolia, euplantulae, claws) are typical for the phasmatodean leg, and thus also for *Sungaya inexpectata* Zompro, 1996, the animal used in this study (Fig. 1). The attachment devices of *S. inexpectata* have been previously investigated individually (Busshardt et al. 2012), as well as acting in concert, showing that (1) they are intended for different surface types and that (2) there exists a synergistic effect between them, i.e., their total attachment performance is greater than the sum of their individual contributions (Büscher and Gorb 2019). *Sungaya inexpectata* possesses smooth arolia, which generate adhesion through large contact areas with the substrate. The euplantulae are structured and bear many little protuberances (this surface is commonly called “nubby” (Büscher et al. 2018)).

In the present study, we were focused on the role of claws, which form a Y-shape together with their tarsal chain. At first glance and according to existing models (Dai et al. 2002), even a single claw should be sufficient to generate grip forces on surface asperities matching their tip sharpness. However, the vast majority of insects evolved two claws. It might be argued that the action of a single claw is enhanced by said Y configuration in which both claws stabilize one another against sideways rotation and associated slipping. We subjected several adult female individuals to gradual amputation of their pretarsal claws and measured their gripping forces on various flat (but differently rough) substrates. This experiment was carried out to gain further insight into how exactly insect claws work in unison with the other tarsal structures, and how their performance on different substrates depends on their count per leg and thus on the tarsus stabilization. The force measurements were conducted in two different directions: perpendicularly from (pull-off) and parallelly along (shear) the substrate. It was hypothesized that loss of claws would decrease attachment performance on all substrates (except the smooth one) to different degrees. On the smooth control surface, we assumed least effect of claws, due to the sufficient adhesive grip by the arolium and due to the lack of appropriately sized surface features to mechanically interlock with.

## Materials and methods

### Animals

Adult females of *Sungaya inexpectata* Zompro, 1996 (Heteropterygidae) were used, to exclude effects based on sexual dimorphism. All animals were sourced from in-house laboratory cultures, kept at a regular day/night cycle and were fed with bramble and oak leaves ad libitum. All experimental animals possessed completely intact legs, claws and attachment pads.

### Substrates

The experiment was conducted using four epoxy resin substrates that were produced using two step molding. They represented molds of polishing papers with different average particle diameters (12 μm, 35 μm and 425 μm), which were chosen because they represent the actual effective roughness range for insect claws (Song et al. 2016). For that range, the largest amount of comparative data from the same taxon exists (Büscher and Gorb 2019). Additionally, a smooth plate made of the same material was used, in order to measure the attachment pad performance only. The two-step molding process was carried out using polyvinylsiloxane-based two component dental wax (President Light Body, Colthéne/Whaledent AG, Altstätten, Switzerland), to obtain the negative surface structure of the polishing papers, which were then filled with epoxy resin (Gorb 2007). Afterwards, these new positives were cured for 24 h at 70 °C. The smooth control substrate was produced in the same way by using a glass plate as template. All substrates measured roughly 20 × 15 cm and were thus suitable to serve as testing surfaces for *Sungaya inexpectata* without it being able to grip at the edge of the substrate.

### Measurements

**Fig. 2:**
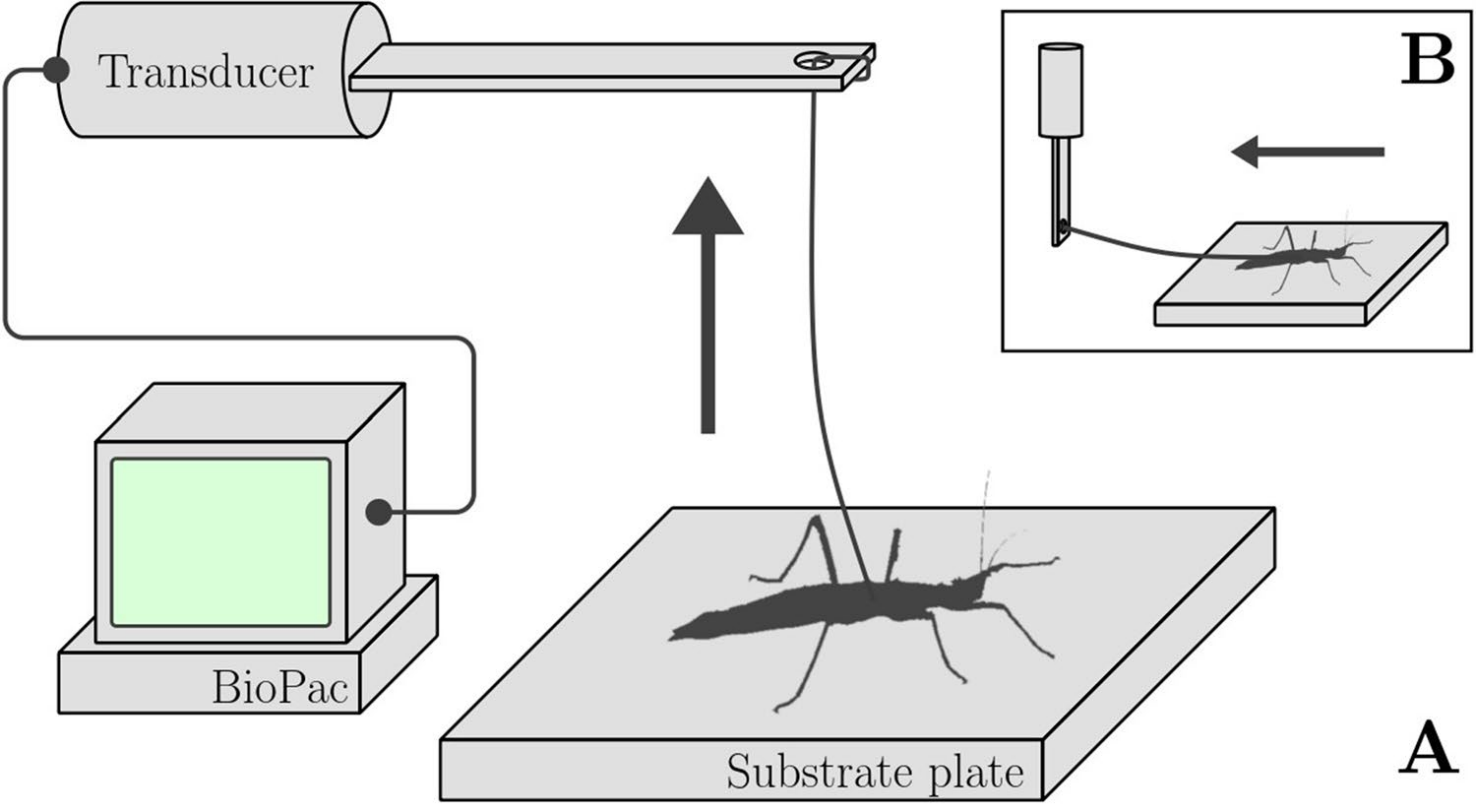
Schematic of the experimental setup. The animal is connected to a force transducer, which in turn is linked to the Biopac data acquisition device. **a** Transducer configuration and acting force direction arrow for pull-off measurements. **b** Transducer configuration and acting force direction arrow for traction measurements

**Fig. 3:**
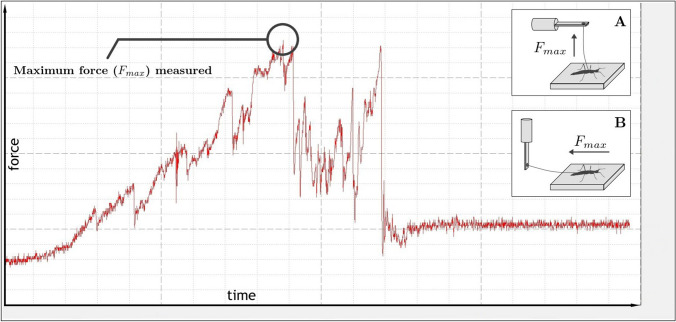
Typical force–time graph created by the program ACQ 3.7.0. Out of each curve, the maximum force value (*F*_max_) was used for later analysis, both for pull-off **a** and traction **b** experiments. This specific curve represents a pull-off measurement. Note that the elevated base value at the end of the curve represents the body weight force of a free hanging individual after pulling it off completely. It was later subtracted from the maximum force

The animals were weighted prior to the measurements using microbalances (AG204 Delta Range, Mettler Toledo, Columbus, OH, USA). They were then connected to a 100 g force transducer (FORT100, World Precision Instruments, Sarasota, FL, USA) by a horse hair tied to their mesothorax, put on one of four (*N*s = 4) substrate plates and either pulled away parallelly (traction measurement), or vertically (pull-off measurement), as is depicted schematically in Fig. 2. For pull-off measurements, the individuals’ body weight force was subtracted from the resultant maximum force. Each measurement for a set of insect individuals, substrate and direction was repeated three times (*N*r = 3). The amplifier MP100 and TCI-102 system (BIOPAC Systems, Inc., Goleta, CA, USA) as well as the software Acqknowledge 3.7.0 (BIOPAC Systems Inc., Goleta, CA, USA) were used to process the signal from the force transducer and to record the corresponding force–time curves (Fig. 3). The whole measurement setup was initially tested for its resolution limit, which yielded an accuracy of ± 7mN.

Furthermore, to document the attachment performance at different numbers of functional claws, the animals were anesthetized using carbon dioxide and their claws were amputated using microscissors. For every complete amputation cycle (all right claws on each leg were amputated, then all left ones), the above measurements were repeated after the animal had recovered after the claw ablation, which took around 15 min. This resulted in six different sets of data—pull-off/traction values for two claws, for one claw, and for zero claws. Later, a final measurement cycle was performed with the same, completely amputated animals after 48 h of recovery, which again yielded two sets of data—pull off and traction. All measurements were conducted in daylight and at ambient temperature (23 °C). The data extraction was performed by identifying the maximum force values (*F*_max_) (Fig. 3) from force–time curves. Then average values, yielding the final force value for that particular animal, claw state, plate and direction, were calculated. The approximate pulling speed was set to 0.5 cm/s using a manually operated moveable *z* stage (pull-off), and x/y stage (traction). *N*_a_ = 11 animals were used for the initial pull-off/traction tests and *N*_a_ = 9 for the tests after 48 h of recovery. The former ones yielded *n* = 132 data points, the latter ones *n* = 108. The legs of the used insects were investigated postmortem after the experiments using scanning electron microscopy (SEM), to rule out damage to the pads during the amputation process. Exemplary images are included in in the supplementary file S2.

### Statistical analysis

For statistical analysis, the program SigmaPlot 12.0 (Systat Software Inc., San José, CA, USA) was used. Data (substrate vs claw number) was tested for normality using a Shapiro–Wilk test, and for equal variance using Levene’s test. The data were afterwards tested for significant relations using two-way analyses of variance (ANOVA) and Holm-Šidák posthoc tests with an alpha value of 0.05. The results of the statistical tests are visualized in the figures and mentioned where of concern, and the test parameters of the ANOVAs are included in the appendix.

### Scanning electron microscopy

Tarsi were cut off from specimens with ablated claws after the attachment force experiments, fixed in 2.5% glutaraldehyde in PBS buffer for 24 h at 4 °C temperature. They were processed in an ascending ethanol series, dried using a Leica EM CPD300 (Leica, Germany) critical-point dried and sputter-coated with 10 nm gold–palladium (Leica Bal-TEC SCD500, Leica, Germany). The tarsi were recorded with a Hitachi S4800 at 5 kV acceleration voltage and a Hitachi TM3000 at 15 kV acceleration voltage (both Hitachi High-technologies Corp., Japan), and checked for damage from the claw amputations.

## Results

The data are reported in the following section in the same format: all force values *F*_*x*_ are the means of all *F*_max_ values obtained in the experiments for the specific claw state, mode and substrate. *M* refers to the corresponding median, *S* to the standard deviation. All statistical statements adhere to the testing procedure outlined in subsection 2.4. Since the inaccuracy incurred by the measurement setup and procedure was ± 7mN, decimals were omitted in all values. This measurement inaccuracy also means that especially values at the lower end of the spectrum are to be viewed increasingly critical, even though the authors are of the opinion that the conclusions drawn from them still hold true, because they follow the trends illustrated by the larger values.

### Traction and pull-off measurements

#### Two claws (intact insects)

**Fig. 4:**
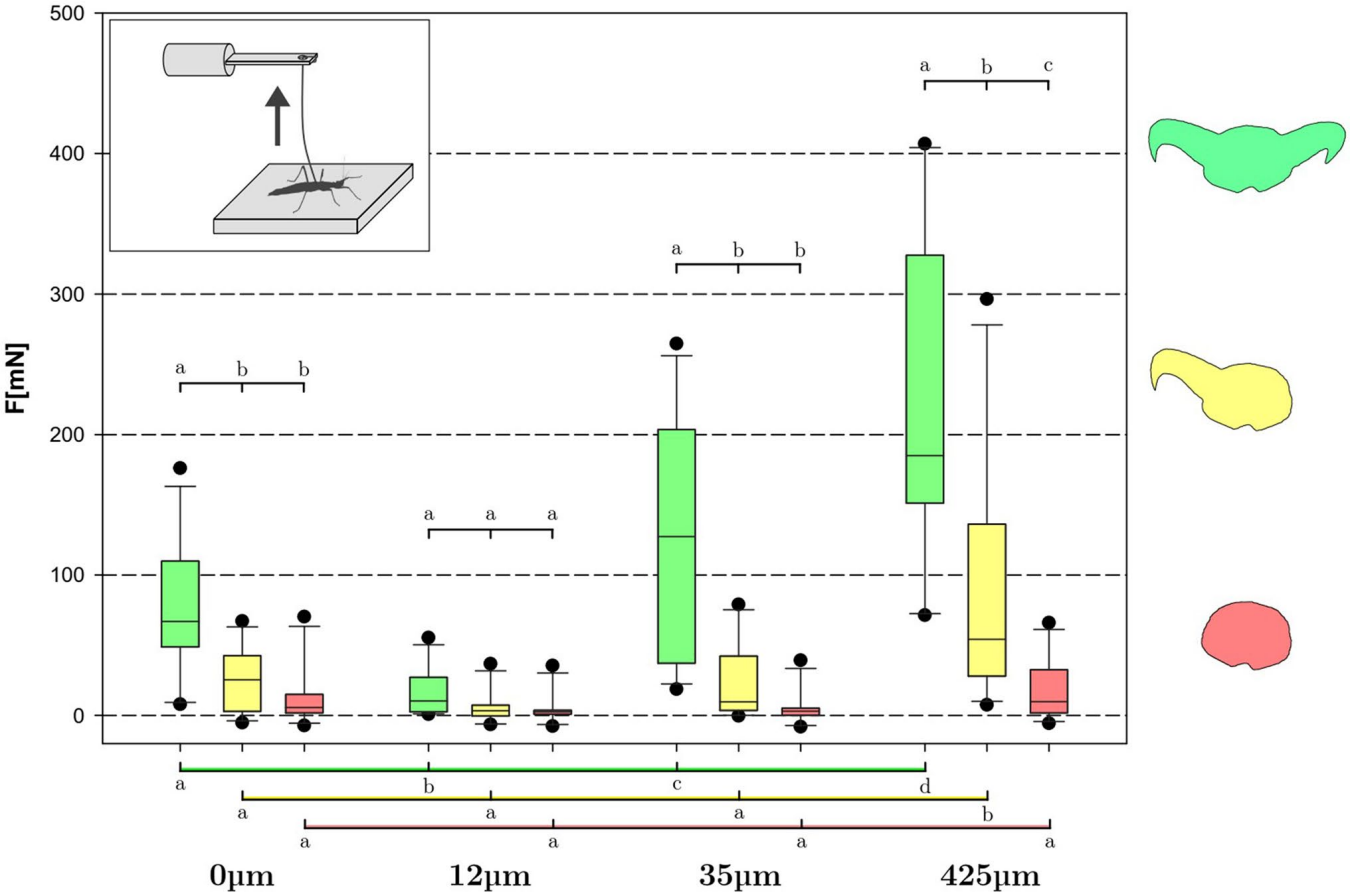
Results of pull-off measurements over all individual insects, substrates and treatments. Attachment performance diminishes with claw number (green = 2, yellow = 1, red = 0). Boxes indicate the 25th and 75th percentiles, whiskers are the 10th and 90th percentiles, the line within the boxes shows the median. Different small letters indicate statistically different (*p* < 0.05) groups within the same brace. N_a_ = 11, *n* = 132, 2-way ANOVA and Holm-Šidák posthoc test

**Fig. 5:**
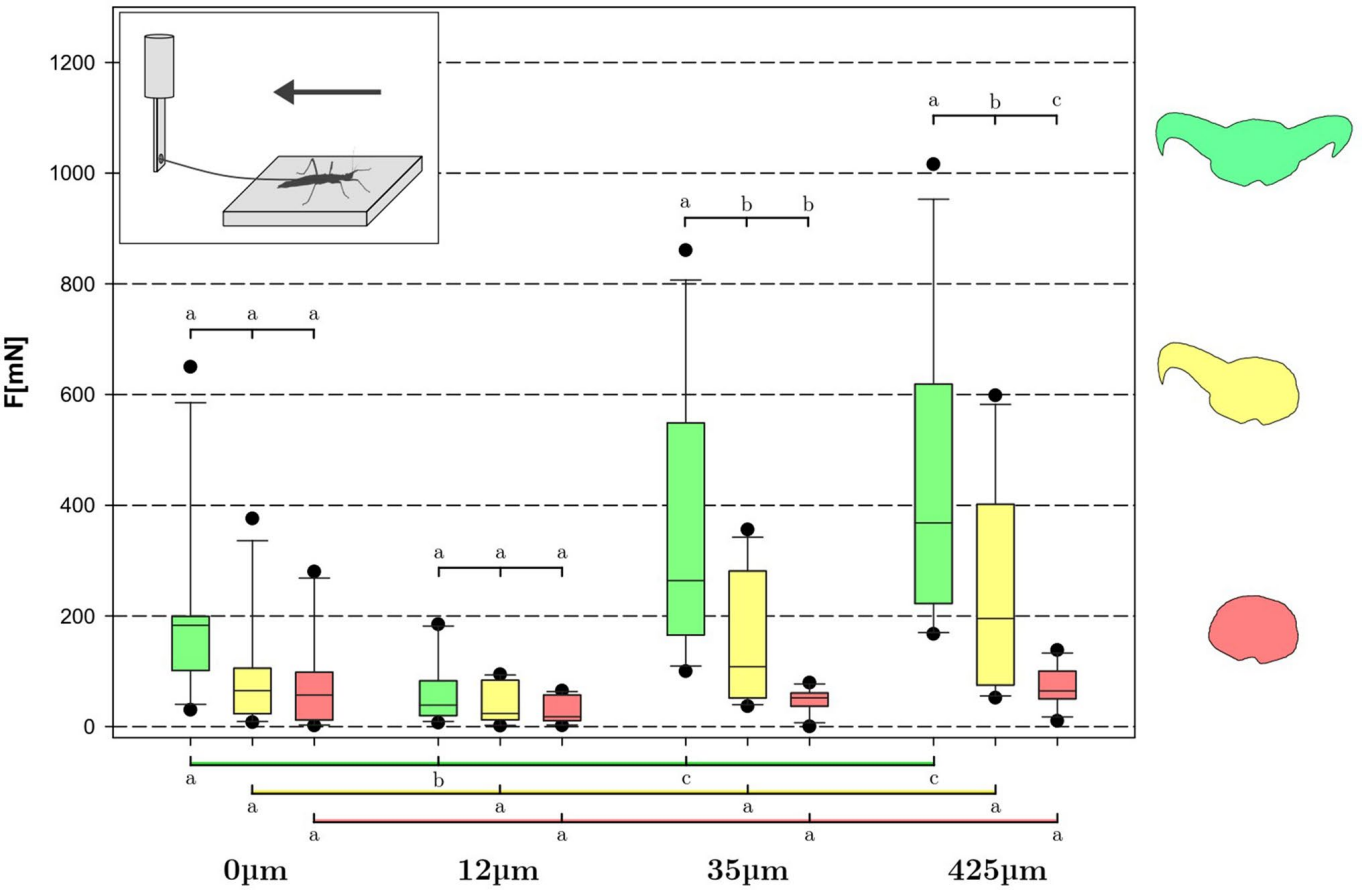
Results of traction measurements over all individual insects, substrates and treatments. Attachment performance diminishes with claw number (green = 2, yellow = 1, red = 0). Boxes indicate the 25th and 75th percentiles, whiskers are the 10th and 90th percentiles, the line within the boxes shows the median. Different small letters indicate statistically different (*p* < 0.05) groups within the same brace. *N*_a_ = 11, *n* = 132, 2-way ANOVA and Holm-Šidák posthoc test

In Figs. 4,5, the data obtained for all insects (*N*_a_ = 11) on all substrates (*N*_s_ = 4) in all claw states (*N*_c_ = 3) is shown for pull-off (Fig. 4) and traction (Fig. 5) experiments, respectively. It became clear that the composite system of claws + arolium had a specific region of substrate roughness at which it worked best in both experiments. For pull-off, performance on 12 μm average particle size was worst (*F*_12_μm = 15 mN, *M* = 11 mN, *S* = 16 mN), while both 35 μm (*F*_35_μm = 126 mN, *M* = 127 mN, S = 77 mN) and 425 μm (*F*_425_μm = 229 mN, *M* = 207 mN, *S *= 111 mN) substrates seemed to provide moderate to high grip for the attachment system. On the smooth substrate (0 μm) (*F*_0_μm = 85 mN, *M* = 72 mN, *S* = 44 mN), the attachment performance notably exceeded that on the 12 μm substrate. Statistically significant differences have been found between all the substrates.

For traction, the results exhibited the same pattern, albeit with larger absolute values (*F*_12_μm = 64 mN, *M* = 46 mN, *S* = 56 mN; *F*_35_μm = 359 mN, *M* = 271 mN, *S* = 221 mN; *F*_425_μm = 434 mN, *M* = 401 mN, *S* = 251 mN; *F*_0_μm = 202 mN, *M* = 185 mN, *S* = 159 mN).

#### Single claw

Animals with a single claw on each leg demonstrated tendencies similar to the intact animals in the pull-off experiment (*F*_12_μm = 5 mN, *M* = 4 mN, *S* = 11 mN; *F*_35_μm = 22 mN, *M* = 11 mN, *S* = 25 mN; *F*_425_μm = 92 mN, *M* = 59 mN, *S* = 85 mN; *F*_0_μm = 23 mN, *M* = 24 mN, *S* = 21 mN). However, the differences between all the substrates have decreased: statistically significant differences have only been found for the pull-off force on 425 μm roughness compared to the other substrates. Traction experiments also did not show any major deviation from this trend (*F*_12_μm = 42 mN, *M* = 33 mN, *S* = 35 mN; *F*_35_μm = 152 mN, *M* = 113 mN, *S* = 111 mN; *F*_425_μm = 242 mN, *M* = 211 mN, *S* = 178 mN; *F*_0_μm = 90 mN, *M* = 71 mN, *S* = 102 mN), and no significant differences have been found between all substrates.

#### No claws

In insects without claws, the differences in pull-off force between substrates almost vanished (*F*_12μm_ = 4 mN, *M* = 3 mN, *S* = 13 mN; *F*_35μm_ = 5 mN, *M* = 3 mN, *S* = 12 mN; *F*_425μm_ = 18 mN, *M* = 13 mN, *S* = 20 mN; *F*_0μm_ = 13 mN, *M* = 6 mN, *S* = 21 mN), even though a minimum force was also obtained at 12 μm substrate. No statistical differences have been found between any of the substrates in this case. This also applies to the traction experiment: even though slightly higher absolute values were obtained, the difference between substrates strongly decreased, and no significant differences were found between them (*F*_12_μm = 28 mN, *M* = 22 mN, *S* = 22 mN; *F*_35_μm = 48 mN, *M* = 50 mN, *S* = 20 mN; *F*_425μm_ = 74 mN, *M* = 67 mN, *S* = 34 mN; *F*_0_μm = 82 mN, *M* = 60 mN, *S* = 85 mN).

### Traction and pull-off measurements after recovery

**Fig. 6:**
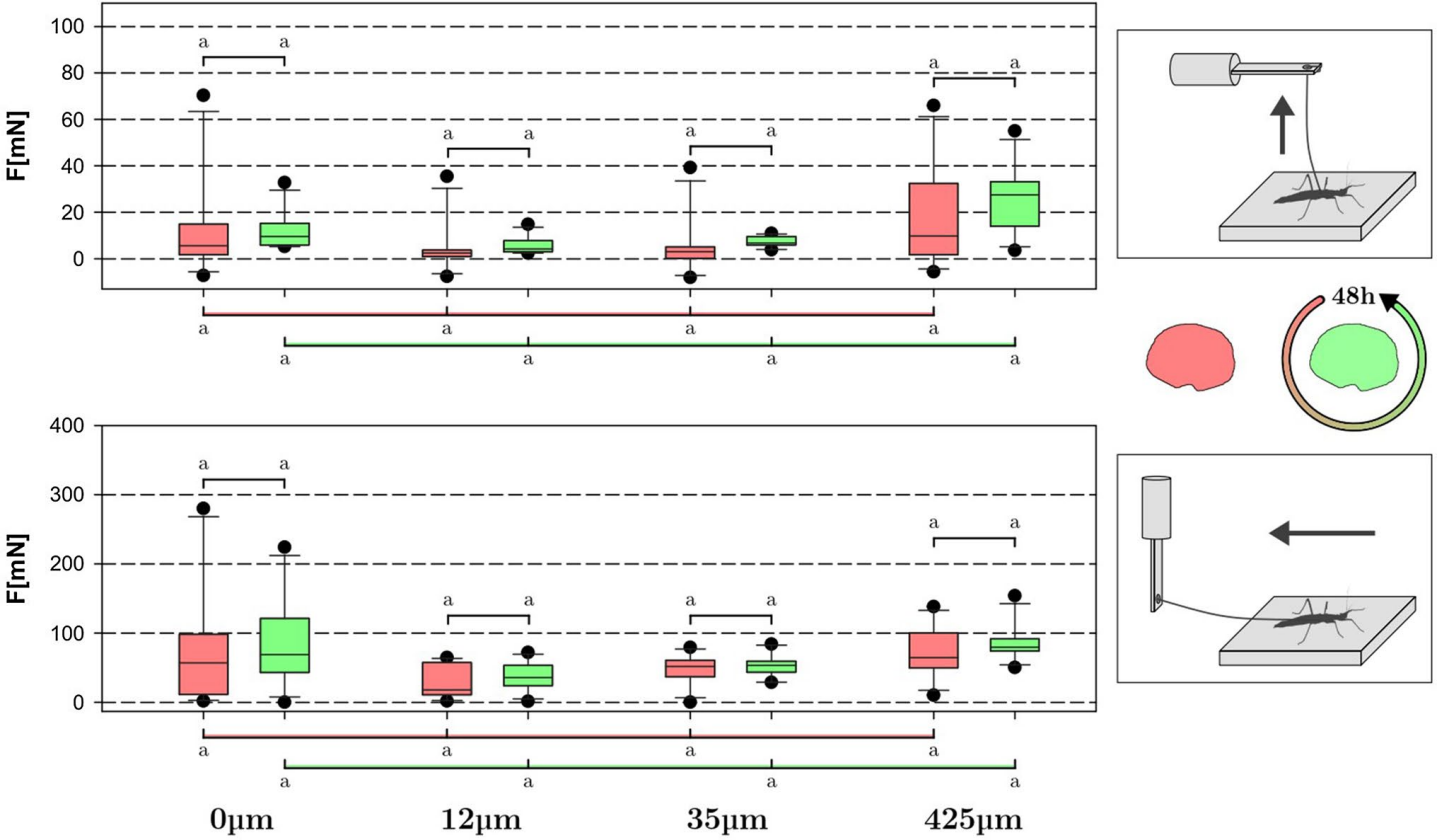
Comparison between exhausted (red) and 48 h-rested (green) animals on all substrates for pull-off and traction. Boxes indicate the 25th and 75th percentiles, whiskers are the 10th and 90th percentiles, the line within the boxes shows the median. Different small letters indicate statistically different (*p* < 0.05) groups within the same brace. *N*_a_ = 9, *n* = 108, 2-way ANOVA and Holm-Šidák posthoc test

Figure 6 shows the results of the follow up measurements using completely claw amputated animals after 48 h of recovery time after the first measurements. The same substrates (*N*_s_ = 4) were used, albeit with two fewer (*N*_a_ = 9) individuals. The data of the initial experiments with animals without claws is included (red) for comparison with the animal performance after recovery time (green). The pull-off results (*F*_12_μm = 6 mN, *M* = 5 mN, *S* = 3 mN; *F*_35_μm = 7 mN, *M* = 7 mN, *S* = 2 mN; *F*_425_μm = 25 mN, *M* = 26 mN, *S* = 14 mN; *F*_0_μm = 12 mN, *M* = 10 mN, *S* = 8 mN) showed no statistically significant recovery effect. Additionally, no significant relationship was found between substrates and the experiment (before and after recovery). Traction measurements likewise did not show any significant recovery effect (*F*_12_μm = 37 mN, *M* = 37 mN, *S* = 19 mN; *F*_35_μm = 53 mN, *M* = 53 mN, *S *= 16 mN; *F*_425_μm = 87 mN, *M* = 83 mN, *S* = 25 mN; *F*_0_μm = 90 mN, *M* = 78 mN, *S* = 60 mN), and no relevant differences between substrates within each experiment (before and after recovery) were found either.

## Discussion

General attachment capabilities of *Sungaya inexpectata* on convex substrates of different diameter (Büscher et al. 2020) and synergistic effects between different attachment devices working in concert (Büscher and Gorb 2019) have been previously studied. In this work, we investigated the functional effect of paired claws on attachment performance on four substrates of different roughnesses. The lowest attachment performance was found on 12 μm substrate under all tested parameters. It can be explained by the fact that neither the adhesion pads nor the claws are suited for this asperity range. This is also in agreement with previous research, which showed that certain roughness regimes are on one side too rough for the attachment pads to get into close contact, and on the other side too fine for the claws to mechanically interlock (Büscher and Gorb 2019; Song et al. 2016). There in any case is a clear connection between surface roughness and claw tip diameter when it comes to attachment performance, which also agrees with previous research (Dai et al. 2002). The exact synergy between insect adhesive pads and claws has been shown to be intricate (Song et al. 2016), and need to be taken into account.

The difference in overall attachment performance between pull-off and traction scenarios can likely be attributed to different possible leg configurations during the experiment. In the pull-off case, the animal tries to adhere to the substrate by placing its feet as spread out as possible, while lowering its body as close to the substrate as it can. This redirects the perpendicular pulling force into the one proximally acting along each leg, so that the claws can interlock with substrate asperities and the whole leg can generate friction forces. However, every increment of the distance between the insect body and the substrate alters the angle between claws and substrate and finally leads to claws disengagement and loss of grip. In contrast, in the traction scenario, the animal can extend all its legs into the direction directly opposing the external force pulling parallel to the substrate. This situation makes claws mechanically interlocking and friction force generation possible without any need of force redirection (Büscher et al. 2020).

After amputation of one claw on each leg, the results changed considerably. Even though tendencies between substrates are preserved, the attachment force medians diminish strongly both for pull-off and traction measurements (Pull-off: 64–91% loss, traction: 28–62% loss). This was somewhat expected, because half of the mechanical interlocking potential was now missing on each leg. Another reason might be that one claw removal destroys the self-stabilizing character of the original claw configuration (two claws and tarsal base forming a Y-shaped contact configuration), making the remaining claw more prone to rotation and slipping off. Especially on rougher substrates, the suboptimal interaction between the adhesion pad and surface does likely not generate enough friction to stabilize the remaining claw against rotational forces (Büscher and Gorb 2019).

After removing all claws, the attachment force medians further diminished on all substrates (Pull-off: 25–78% loss, traction: 15–68% loss). It has to be noted that a sizeable portion of the values obtained in this part of the experiment is of the same order of magnitude as the sensor noise as described in section 2.3. This does mean that these values have to be viewed more critically than the larger ones obtained in the previous measurements. However, the sensor noise’s oscillation period is extremely small (on the order of ms) with respect to the measurement speed and -duration per pulling (0.5 cm/s over 20 s on average). Additionally, multiple animals have undergone multiple repetitions of the same measurement, averaging the possible influence of the noise. Therefore, it stands to reason that the relative magnitudes of these values are still approximately correct because the noise can be expected to have converged towards a common offset between the measurements. This means that while the absolute values can be expected to be skewed slightly by said offset, the relations between the same types of measurements should be representative. Since the conclusions drawn from those values are based on those relations rather than absolute values, they thus should still hold. This viewpoint is further supported by that fact that the overall tendency found with the initial experiments is preserved.

On the roughest substrate (425 μm), animals were still able to attach moderately. This was likely possible due to the fact that the remaining claw stumps were able to find some grip there, even with their strongly increased contact tip diameter. The tibial spurs, situated on the most distal part of the tibia, may potentially have generated some friction as well, but likely not in a significant way due to their distal orientation. Spurs pointing in this direction would be able to produce friction when resisting distal pulling, but the forces the legs needed to resist in our experiments mainly acted proximally along them. During all traction experiments, the animals only ever oriented all of their legs into the direction opposed to the pulling. Therefore, even the hind legs should not have contributed in a significant way, even though their spurs would have been facing the correct direction in the legs’ normal orientation. The role of tibial spurs has been investigated in the beetle *Pachnoda marginata* (Drury, 1773) (Scarabaeidae), and it was found that they were neither used for clinging to rough surfaces nor walking on flat ones, but rather for generating propulsion by interlocking with the surface during locomotion in narrow gaps (Busshardt et al. 2014). Following this, a contribution of the spurs to the overall force is likely, however, the effect should have been small.

Interestingly, attachment performance strongly diminished even on the smooth substrate in the course of both amputation cycles. This is notable, as on a smooth surface, the claws should not be able to contribute significantly to the attachment performance, because no appropriately sized surface asperities exist to interlock with. An inaccurate amputation and damage of attachment pads during claw ablation were ruled out by the subsequent SEM investigation (see supplementary file S2). The additional measurements after 48 h recovery time were conducted to eliminate possible exhaustion effects elicited by prolonged force measurements. The results, however, suggest little to no overall recovery effect on the attachment performance on the smooth substrate, even though some values seem to suggest otherwise (most prominent example: *M* = 60 mN for traction with no claws without 48 h recovery, *M* = 78 mN for traction with no claws with 48 h of recovery, both on 0 μm substrate). There is a number of possible explanation for this outlier, but since no statistically significant differences have been found, and because the recovery effect, if present at all, does not even remotely restore the attachment performance on smooth substrate to unamputated levels, it can likely be ignored in context of this work’s conclusion: there indeed exists a direct relationship between the attachment pad performance on smooth substrate and the presence of intact claws. It might be possible that the intact Y-shaped claw configuration stabilizes the attachment pad between the claws against rotation and sideway bending that would possibly diminish pad contact area with the substrate due to wrinkling and strain. Other arthropods, as for example representatives of Hymenoptera, possess internal sclerite-like structures called arcus inside their arolia, which provide mechanical stability for the attachment pad and enable its use independently of claws (Frantsevich and Gorb 2002; Gladun 2008; Federle et al. 2001). Thysanoptera, as another example, do not possess pretarsal claws as such, but still feature pretarsal attachment pads, albeit with a very different (dis-) engaging mechanism (Heming 1972). Both examples suggest the necessity of intact, sufficiently large claws for reliable attachment pad performance in absence of internal stabilization structures. Taking this into account, it seems reasonable to attribute the observed effect to the lack of such an internal structure in the phasmatodean attachment pads, which can crumble without sufficient mechanical support by paired claws.

Overall, the results of this study are comparable to some other works, in which insects have been subjected to claw amputation, as removal of claws generally leads to loss of attachment performance and/or propulsion generation on flat, appropriately rough surfaces. In some instances, the results suggest a certain interplay between different attachment devices. Salerno et al. have shown that ablation of claws in the stinking bug *Nezara viridula* (Linnaeus, 1758) (Pentatomidae) reduced attachment performance not only on rough substrates, where it was to be expected, but also on smoother ones (Salerno et al. 2018). Pattrick et al. have shown that insects face a trade-off between claw tip sharpness and claw fragility – sharper tips better interlock with surface asperities, but are more likely to break and bend elastically, which also makes slipping more likely. This is especially true if there is no second claw or other structures to mechanically stabilize it (Pattrick et al. 2018). This ties in with results of other works by Song et al. and Dai et al. who, among other things, showed that claws tend to be less mechanically stable on certain roughnesses (Song et al. 2016; Dai et al. 2002). It therefore stands to reason that this effect is more pronounced with single claws that are not stabilized by any other structure. Theunissen and Dürr (2013) proved that the stick insect *Carausius morosus* (Brunner von Wattenwyl, 1907) (Lonchodidae) takes two different kinds of walking step sizes depending on the geometric difficulty of the terrain (flat ground vs. steps of different height, all smooth polyvinyl chloride), and proposed that those shorter steps lead to safer locomotion in those scenarios. After ablating the claws, they were able to show that the proportion of small walking steps in the insects’ overall movement increased, implying that the claws were playing a role in providing adequate footholds even on smooth surfaces.

Taking all of this into account, the reason why insects for the vast majority possess two pretarsal claws instead of one or three can likely be broken down into different aspects:

1. A pair of claws provides two points of contact and interlocking with a substrate, which self-stabilizes the system against rotation, slipping and bending that would more likely occur with only one claw. This advantage would also exist for three claws, but it would reduce the leg’s target asperity range because three claw tips constitute a plane instead of the line two tips create. This is relevant because three tips would have more difficulty in finding three appropriate surface features to interlock with that are still located within said plane—the leg would effectively lose a degree of rotational freedom in choosing target features. While this problem can be alleviated by making the individual claws more mobile, the increased interlocking strength likely doesn’t justify the additional biological investment.
2. In addition to the benefits of self-stabilization and relative flexibility in the asperity target range, two claws still leave space for other specialized attachment devices like attachment pads in between them, which would be more difficult to incorporate with a third claw. With two claws, the spatial distribution of those different structures remains clear. Their operational zones do not overlap and they do not interfere with each other’s function.
3. The fact that two claws leave space in between them also increases the efficiency of the arolium: since claws on both sides stabilize the attachment pad, it is less likely to collapse, wrinkle and/or rotate, all of which would decrease its contact area and thus its performance. While in a one claw system this kind of stabilization can also be achieved by internal sclerites inside the arolium or specialized movement abilities, those constitute additional biological investments that are not required in a two claw configuration.

## Conclusions and outlook

In this paper, we studied the effect of gradual removal of pretarsal claws on the attachment performance in the stick insect *Sungaya inexpectata*. It was observed that in insects with ablated claws, the generated attachment force on rough substrates decreased, but also, notably, on a smooth one. This effect was unexpected, since smooth substrates lack appropriately sized surface features for the claws’ mechanical interlocking. Considering this, we conclude that there is a direct connection between the existence of intact claws and the performance of attachment pads. We propose that this effect is due to the absence of mechanically stabilizing internal structures within the pretarsal arolium, and that the arolium performance depends on the external stabilization through the paired symmetrical claws. This study provides some important design rules for further use in the robotics for an optimized construction of biologically-inspired terminal effectors of robotic legs for gripping on the broad variety of substrates. Further investigation of the biomechanic interplay between claws and arolia is necessary to understand the exact mechanism of self-stabilization of the pretarsus and arolia in contact with various substrates.

## Supplementary Information

Below is the link to the electronic supplementary material.

Supplementary file1 (DOCX 2126 KB)
