# Supplementary material for: Gripping performance in the stick insect Sungaya inexpectata in dependence on the pretarsal architecture
Source: J Comp Physiol A Neuroethol Sens Neural Behav Physiol. 2022 Sep 24;209(2):313–23. doi: 10.1007/s00359-022-01570-1 (PMC10006028; doi:10.1007/s00359-022-01570-1)
Supplement: Supplementary file 1 — Supplementary file1 (DOCX 2126 KB) [file 359_2022_1570_MOESM1_ESM.docx]

9 Appendix

Supplementary file S1. Tabellaric data for statistical analyses

The tables below represent the results of the 2-way ANOVAs.

| Pull-off | | | | | |
| --- | --- | --- | --- | --- | --- |
| Source of variation | DF | SS | MS | F | P |
| # Claws | 2 | 244570.236 | 122285.118 | 43.919 | <0.001 |
| Substrate | 3 | 194188.217 | 64729.406 | 23.247 | <0.001 |
| # Claws x Substrate | 6 | 129639.739 | 21606.623 | 7.760 | <0.001 |
| Residual | 120 | 334123.401 | 2784.362 |  |  |
| Total | 131 | 902521.594 | 6889.478 |  |  |

| Traction | | | | | |
| --- | --- | --- | --- | --- | --- |
| Source of variation | DF | SS | MS | F | P |
| # Claws | 2 | 970479.788 | 485239.894 | 25.713 | <0.001 |
| Substrate | 3 | 759022.575 | 253007.525 | 25.713 | <0.001 |
| # Claws x Substrate | 6 | 406972.069 | 67828.678 | 3.594 | 0.003 |
| Residual | 120 | 2264589.580 | 18871.580 |  |  |
| Total | 131 | 4401064.012 | 33595.908 |  |  |

| Pull-off exhausted/rested | | | | | |
| --- | --- | --- | --- | --- | --- |
| Source of variation | DF | SS | MS | F | P |
| # Claws | 2 | 295635.763 | 147817.882 | 72.726 | <0.001 |
| Substrate | 3 | 117747.226 | 39249.075 | 19.311 | <0.001 |
| # Claws x Substrate | 6 | 159178.530 | 26529.755 | 13.053 | <0.001 |
| Residual | 120 | 243902.138 | 2032.518 |  |  |
| Total | 131 | 816463.657 | 6232.547 |  |  |

| Traction exhausted/rested | | | | | |
| --- | --- | --- | --- | --- | --- |
| Source of variation | DF | SS | MS | F | P |
| # Claws | 2 | 1209346.817 | 604673.408 | 42.451 | <0.001 |
| Substrate | 3 | 421891.250 | 140630.417 | 9.873 | <0.001 |
| # Claws x Substrate | 6 | 521881.448 | 86980.241 | 6.106 | <0.001 |
| Residual | 120 | 1709290.650 | 14244.089 |  |  |
| Total | 131 | 3862410.165 | 29484.047 |  |  |

Supplementary file S2


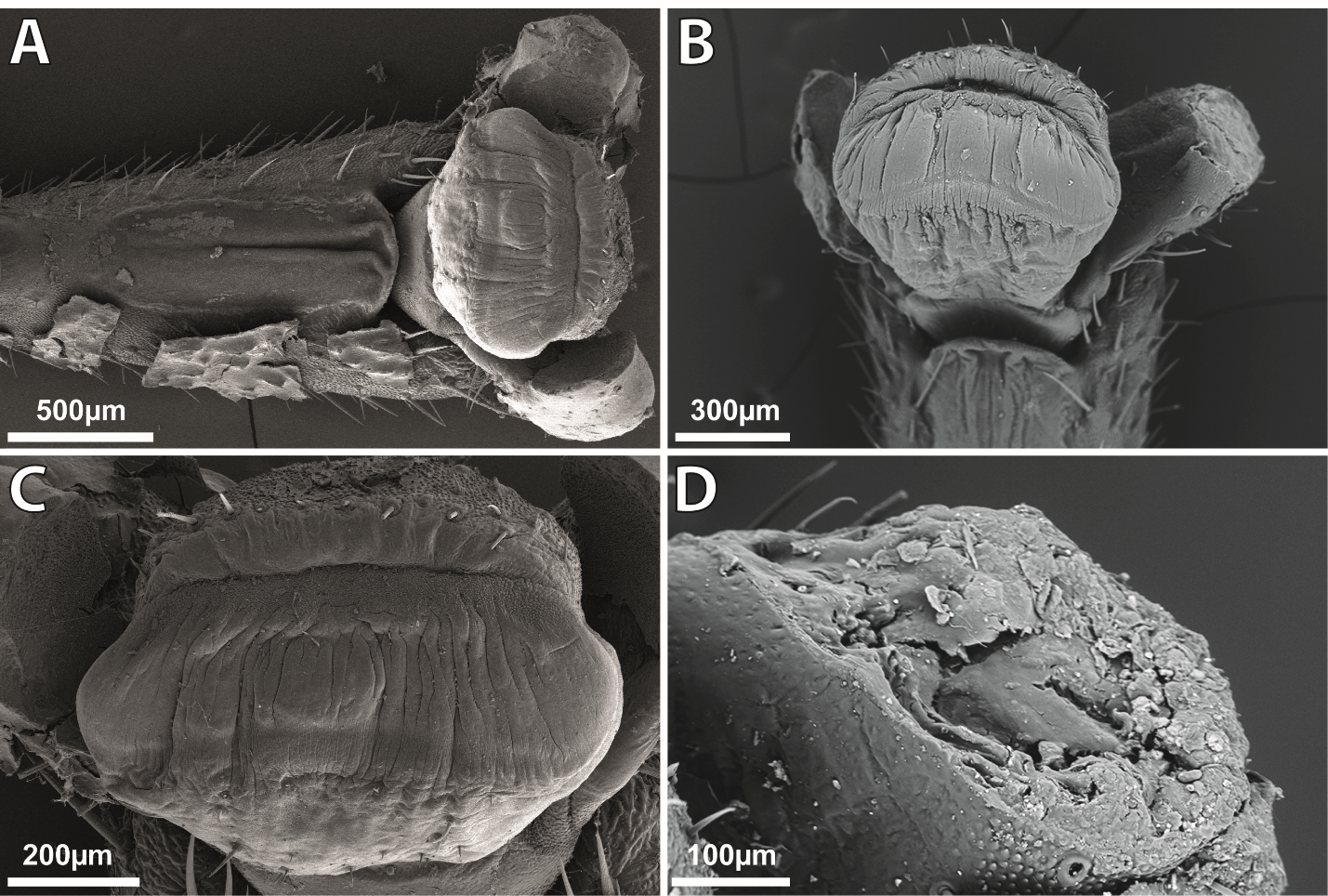


**Fig. S1** SEM images tarsi after claw ablation and experiments. (A, B) Overview of ablation sites. (C) Intact arolium. (D) Cut surface of sealed claw residual.
